# An Unsupervised Machine Learning Approach for Monitoring Data Fusion and Health Indicator Construction

**DOI:** 10.3390/s23167239

**Published:** 2023-08-18

**Authors:** Lin Huang, Xin Pan, Yajie Liu, Li Gong

**Affiliations:** 1 Ship Comprehensive Test and Training Base, Naval University of Engineering, Wuhan 430033, China; huanglin2038@163.com (L.H.);; 2 School of Electrical Engineering, Naval University of Engineering, Wuhan 430033, China

**Keywords:** health indicator, monitoring data, machine learning, autoencoder

## Abstract

The prediction of system degradation is very important as it serves as an important basis for the formulation of condition-based maintenance strategies. An effective health indicator (HI) plays a key role in the prediction of system degradation as it enables vital information for critical tasks ranging from fault diagnosis to remaining useful life prediction. To address this issue, a method for monitoring data fusion and health indicator construction based on an autoencoder (AE) and a long short-term memory (LSTM) network is proposed in this study to improve the predictability and effectiveness of health indicators. Firstly, an unsupervised method and overall framework for HI construction is built based on a deep autoencoder and an LSTM neural network. The neural network is trained fully based on the normal operating monitoring data and then the construction error of the AE model is adopted as the health indicator of the system. Secondly, we propose related machine learning techniques for monitoring data processing to overcome the issue of data fusion, such as mutual information for sensor selection and t-distributed stochastic neighbor embedding (T-SNE) for operating condition identification. Thirdly, in order to verify the performance of the proposed method, experiments are conducted based on the CMAPSS dataset and results are compared with algorithms of principal component analysis (PCA) and a vanilla autoencoder model. Result shows that the LSTM-AE model outperforms the PCA and Vanilla-AE model in the metrics of monotonicity, trendability, prognosability, and fitness. Fourthly, in order to analyze the impact of the time step of the LSMT-AE model on HI construction, we construct and analyze the system HI curve under different time steps of 5, 10, 15, 20, and 25 cycles. Finally, the results demonstrate that the proposed method for HI construction can effectively characterize the health state of a system, which is helpful for the development of further failure prognostics and converting the scheduled maintenance into condition-based maintenance.

## 1. Introduction

In the field of Prognostics and Health Management (PHM) [1], real-time prediction of equipment degradation is crucial as it serves as an important basis for developing Condition-Based Maintenance (CBM) strategies [2]. However, in most cases, the data measured by the monitoring system cannot track the degradation status of the system. Therefore, a model that can predict system degradation or a quantifiable indicator is needed to evaluate the health status of the system, such as the system health indicator (HI). HI reflects the current health status of the system and is an important basis for predicting the trend of system fault development and performance degradation, which can be used to estimate the time when the system reaches failure [3]. Therefore, constructing a reasonable and accurate system HI is an important foundation for developing CBM strategies. For example, HI plays an important role in anomaly detection [4], remaining useful life (RUL) prediction [5], and health state prediction [6], and the effectiveness of the implementation in these fields heavily depends on HI construction [7].

The construction of HI can be divided into two types: model-based methods and data-driven methods, or directly using parameters with clear physical meanings in the system, such as the crack length of mechanical devices and the vibration amplitude of rotating machinery. Among these, model-based methods require in-depth analysis of system characteristics and the establishment of physical failure models based on this, which usually requires rich domain knowledge [8]. Currently, with the development of data collection and storage technology, data-driven methods have become the mainstream method for constructing HI. The core idea is to fuse multiple measurement data to obtain the comprehensive HI of the system.

The evaluation of system health status can be summarized into two questions: what data to use, and how to process and analyze the data. Usually, the most easily obtained data for a system are its monitoring data, which are typically time series data. The monitoring data of the system actually reflect many characteristics of the system, and by deeply mining the system monitoring data, a large amount of relevant information about the system’s health status can be obtained. For example, in [9], several data-driven models have been proposed and applied for diagnostics and prognostics purposes in complex systems based on monitoring data. In [10], a PHM model for the prediction of component failures and the system lifetime is proposed by combining monitoring data, time-to-failure data, and background engineering knowledge of the systems. Likewise, in [11], the degradation index of a rolling bearing is constructed based on the monitoring data.

Regarding the issue of HI construction, a certain number of researchers have adopted traditional machine learning methods such as principal component analysis [12], quantitative programming [13], support vector machines [14], hidden Markov model [15], and Bayesian linear regression [16]. In recent years, with the massive accumulation of data and the development of computer hardware, as an important branch of machine learning, deep learning technology has been rapidly developed. Currently, deep learning techniques have been applied to HI construction in relevant literature; these techniques include vanilla neural networks [17], recurrent neural networks (RNNs) [18], long short-term memory networks [19,20], convolutional neural networks [21,22], and generative adversarial networks [23].

Among the current numerous deep learning methods, an autoencoder is a kind of unsupervised learning method, which is essentially a custom-built neural network architecture. The architecture of the autoencoder consists of a decoder and an encoder. It was first proposed by Rumelhart et al. and is mainly used for data dimension reduction [24]. A deep autoencoder is a multi-layer feedforward neural network with narrow middle and wide ends. It compresses the input data into a low dimensional projection through the encoder, and then reconstructs the output data through the decoder, trying to make them similar enough. Thus, the dimension of input data can be reduced in a hierarchical manner, achieving high-quality data reconstruction. With the development of autoencoder methods, derivative methods such as the denoising autoencoder [25], sparse autoencoder [26], and stacked autoencoder [27] have been invented. In the field of PHM research, the autoencoder has also been gradually applied to anomaly detection [28,29], RUL prediction [30], fault diagnosis [31,32], and health status assessment [33].

For HI construction, we use a basic assumption that when the system’s health status deteriorates, the monitoring data of the system will gradually deviate from the range of normal data, and the more the system performance degrades, the greater the degree of deviation. Therefore, the autoencoder can successfully reconstruct normal data samples after training, but for the abnormal data of the system and the monitoring data after system degradation, an autoencoder model is expected to obtain a large reconstruction error, and the more serious the system degradation, the larger the reconstruction error should be. Based on this, it is reasonable to use autoencoder to build a system HI based on system monitoring data. However, as mentioned earlier, the monitoring data of the system are typically time series data, and methods mentioned above do not fully consider the time series characteristics of monitoring data and the correlation between variables. The LSTM neural network is a kind of recurrent neural network that is specially designed to solve the vanishing gradient problem existing in general RNNs. It can learn the relationship between variables in time series, capture the time series characteristics of monitoring data, and better realize the encoding and decoding of time series data.

By comparison, for time series analysis, sensor selection, also known as feature selection, is one of the important factors that determine the final effect, which plays a vital role in the quantitative analysis of data. At present, for the feature analysis of the time series of the HI, in most cases, the selection of variables is mainly undertaken manually by observing the trend, amplitude, noise, and other characteristics of the variables. This approach has problems such as strong subjectivity, inability to conduct quantitative analysis, and inaccuracy [34]. To solve these problems, a sensor selection method based on mutual information (MI) is proposed to determine the input data of the autoencoder. Mutual information is a measure of the interdependence between two variables, and indicates how much information is shared between two variables [35]. The greater the mutual information between two variables, the stronger their correlation [36]. It has become one of the important methods in feature selection for calculating and analyzing the mutual information of features, judging the correlation between features, and then selecting the features with strong correlations with target variables [37]. However, for feature extraction and analysis of time series, one of the main problems of mutual information-based methods is identifying suitable target variables; that is, the key to mutual information-based methods is to identify appropriate target data. In most cases, there is no intuitive and directly available target information and the existing literature and research are mainly focused on the improvement in the feature extraction algorithm based on mutual information itself. To solve the above problems, we propose a target information extraction method based on PCA dimensionality reduction. On this basis, a mutual information method coupled with target variables is constructed to distinguish and extract applicable monitoring sensor data.

In view of the above situation, we propose an HI construction method based on a deep autoencoder, which is used to assess system health status based on high-dimensional monitoring time series data. The model is composed of a neural network composed of an encoder and a decoder, and LSTM is used to optimize the autoencoder network structure. While extracting the information of monitoring time series data, the method can complete the bidirectional mapping of data between the high-dimensional feature space and the low-dimensional latent space. The autoencoder compresses the time series into a low-dimensional space, and then uses the decoder to reconstruct the compressed data, minimize the reconstruction error, and construct the HI of the system by the degree of the reconstruction error.

### 1.1. Highlight and Contribution

To summarize, we propose an HI construction method based on LSTM and autoencoder neural networks for the health assessment of complex systems. This method is a data-driven approach that uses system health operating data to train neural networks, and uses the reconstruction error of the decoder as the basis for HI construction. To verify the effectiveness of the method, we conduct HI construction experiments based on the CMAPSS dataset and compared with the most widely used PCA dimensionality reduction method for extracting HI from multiple metrics. In summary, the contributions of this research are as follows:

(1) We propose a construction method for a system HI based on an autoencoder and LSTM neural network. As the system gradually degrades and reaches failure, the monitoring data will gradually deviate from the normal range. Based on this assumption, the LSTM neural network is used to obtain the data series characteristics of the system monitoring data, and the system health index is constructed according to the reconstruction error of the autoencoder.

(2) We design a specific technical process for implementing HI construction based on the above theoretical methods. In this process, we focus on data processing of sensor selection and operating condition identification, and propose a sensor selection method based on mutual information and operating condition identification method based on t-distributed stochastic neighbor embedding.

(3) The effectiveness of the proposed method is verified based on a publicly available aviation engine operation dataset. The experimental results are compared with other methods to verify the feasibility of the proposed method. Through qualitative and quantitative analysis, it is shown that the proposed method does not require the establishment of an accurate mathematical model for the system, and can effectively achieve the health status assessment of the system.

### 1.2. Organization

The remainder of this paper is organized as follows. In Section 2, we describe the architecture of the proposed system and introduce the theoretical foundation of the methodology. Results of the experiments are presented and discussed in Section 3. Finally, the conclusions and future work are set out in Section 4.

## 2. Methodology

### 2.1. Overall Framework

**Figure 1:**
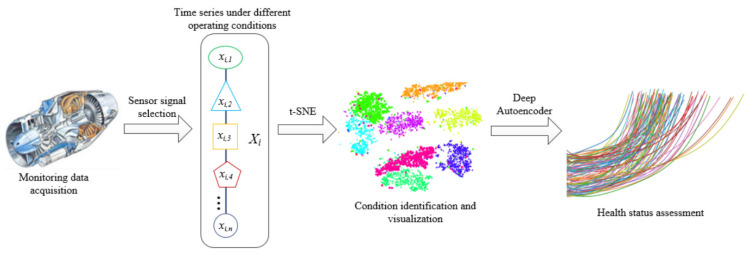
Autoencoder-based health status assessment process (The different colors mean different working conditions, being separated by the algorithm).

The key to conducting system health assessment is to establish a system health assessment model, that is, to establish a mapping relationship between multidimensional monitoring variables and the system HI. In the actual process, the system will operate alternately under different working conditions, and due to the different operating states of the system, there may be significant differences in the monitoring data of the system. Furthermore, under different operating conditions, the degradation rate and failure threshold of the system also vary, which will increase the difficulty of evaluating the health status. Based on the idea of divide and conquer, a system HI construction method and process, as shown in Figure 1, are proposed for the problem of system health assessment in multiple working conditions. This process includes the following main steps: Step 1: select sensor data for health assessment that contain rich information and can reflect system degradation; Step 2: divide the working conditions based on sensor data and classify the data samples according to different working conditions; Step 3: establish health assessment models based on the autoencoder for different working conditions and calculate the comprehensive HI; Step 4: integrate the comprehensive HI of all monitoring data in chronological order to obtain the system degradation curve in the form of HI sequences.

### 2.2. Deep Autoencoder

**Figure 2:**
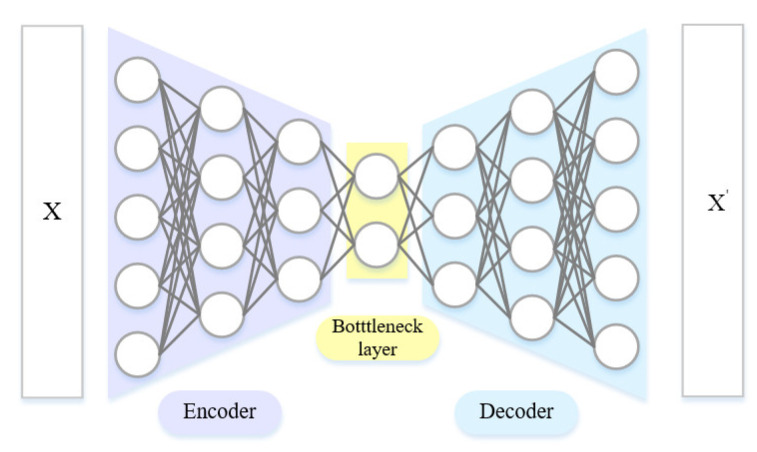
Deep autoencoder.

An autoencoder is an unsupervised neural network with training in two stages. It can simplify high-dimensional data into low-dimensional data, and then reconstruct low-dimensional data into high-dimensional input vectors. In the process, the reconstructed high-dimensional vectors are made as close to high-dimensional input vectors as possible. On this basis, a deep autoencoder builds a deep model through superposition. The hidden state of the upper layer is taken as the input of the lower layer. The model is trained with greedy learning layer by layer. Its basic structure is shown in Figure 2. In this case, the encoder is used to transform the high-dimensional data in the input layer into the low-dimensional data in the hidden layer. Meanwhile, the decoder reconstructs the low-dimensional data in the hidden layer into the high-dimensional data in the output layer. Through constant training, the autoencoder structure can deliver reconstructed output that is approximate to the original input. This is mainly intended to denoise the data and extract its truly useful features.

When the data X are input into the encoder, they are transformed by dimension reduction layer by layer until the bottleneck layer is reach, because the number of nodes in the encoder’s neural network decreases layer by layer. Next, the decoder is used to expand such data into $X^{\prime }$. The difference between the input X and the output $X^{\prime }$ is taken as the optimization objective function to adjust the parameters of the neural network, so as to guarantee their approximation as much as possible. To be specific, the input data are assumed to be X, so that the output of the encoder is:

(1) $$h(X)=\sigma \left(W^{\left(EN\right)}X+b^{\left(EN\right)}\right)$$

where *σ* is the encoder activation function, and *W*^(*EN*)^ and *b*^(*EN*)^ are the encoder’s neural network weight matrix and bias vector, respectively. The output of the decoder is:

(2) $$X^{\prime }=\sigma \left(W^{\left(DE\right)}h(X)+b^{\left(DE\right)}\right)$$

where *W*^(*DE*)^ and *b*^(*DE*)^ are the decoder’s neural network weight matrix and bias vector, respectively. To the autoencoder as a whole, the reconstruction error to be minimized can be expressed as:

(3) $$D(X,X^{\prime })=\|X-X^{\prime }{\|}_{2}$$

While searching for the parameters needed to minimize the reconstruction error, the autoencoder can gradually adjust the parameters of the network to achieve the permissible convergence value of the reconstruction error, that is:

(4) $$W^{\left(EN\right)},b^{\left(EN\right)},W^{\left(DE\right)},b^{\left(DE\right)}=\arg\min\|X-X^{\prime }{\|}_{2}$$

### 2.3. Long Short-Term Neural Network

**Figure 3:**
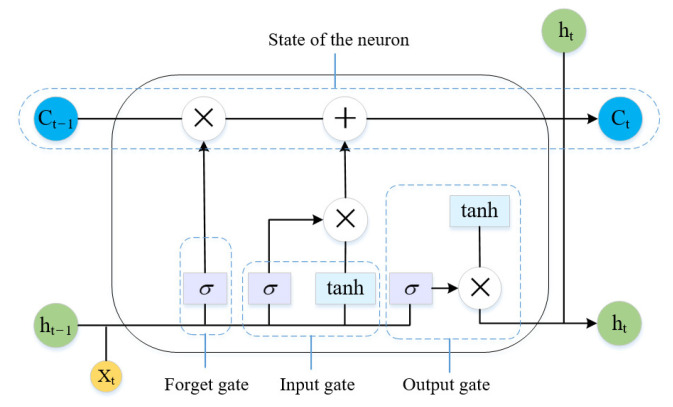
Structure of a long short-term memory network unit.

Long short-term memory (LSTM) is a special type of a recurrent neural network. With the series data as its input, an RNN recurs in the evolution direction of a series, and has all nodes linked. Normally, it is trained by a back propagation through time (BPTT) algorithm. Nevertheless, the inference rules of a BPTT algorithm make it very easy for an error gradient to cause nonlinear behaviors such as vanishing or exploding gradients after the back propagation of multiple time steps. The LSTM neural network relies on gate control to combine short- and long-term memories, which resolves the problem of a vanishing gradient to some extent [38]. The internal structure of an LSTM neural network unit is given in Figure 3.

An LSTM unit consists of three gates, that is, input, forget, and output. The input and output gates are used to control the input and output activation of the memory unit, respectively. The forget gate can update the state of the unit. The module formed by these gates guarantees that the error travels in the form of a constant instead of a product in the network. This can resolve the problem of a vanishing or exploding gradient. The parameters of the unit are updated by the following equations:

(5) $$f_{t}=\mathrm{sgmoid}\left(W_{xf}\cdot x_{t}+W_{hf}\cdot h_{t-1}+b_{f}\right)$$

(6) $$i_{t}=\mathrm{sgmoid}\left(W_{xi}\cdot x_{t}+W_{hi}\cdot h_{t-1}+b_{i}\right)$$

(7) $${\overset{⌣}{C}}_{t}=\tanh\left(W_{xa}\cdot x_{t}+W_{ha}\cdot h_{t-1}+b_{a}\right)$$

(8) $$o_{t}=\mathrm{sgmoid}\left(W_{xo}\cdot x_{t}+W_{ho}\cdot h_{t-1}+b_{o}\right)$$

(9) $$C_{t}=f_{t}⊗C_{t-1}+i_{t}⊗{\overset{⌣}{C}}_{t}$$

(10) $$h_{t}=o_{t}⊗\tanh\left(C_{t}\right)$$

where *h_t_*_−1_ and *C_t_*_−1_ are the output and the state of the unit at the previous time; *x_t_* is the input at the current time; *f*, *i*, and *o* are the forget, input, and output gates, respectively; *f_t_* is the forget control signal for the state of the unit at the previous time; *f_t_*⊗*C_t_*_−1_ is the information kept at the previous time; ${\overset{⌣}{C}}_{t}$ is the state of the candidate unit at the current time; *i_t_* is the control signal of ${\overset{⌣}{C}}_{t}$; *h_t_*_−1_ is the final output; *o_t_* is the output control signal; *W_x_* is the link matrix of input and hidden layers; *W_h_* is the link matrix of output and hidden layers; and ⊗ is the dot product between matrices.

### 2.4. Sensor Selection Based on Mutual Information

Mutual information is a measure of the interdependence between two variables, which mainly represents the correlation between two variables. Assuming that the joint probability distribution of two variables X and Y is $p\left(x,y\right)$, and their marginal probability distributions are $p\left(x\right)$ and $p\left(y\right)$, respectively, then mutual information $MI\left(X,Y\right)$ is the relative entropy of the joint probability distribution $p\left(x,y\right)$ and the marginal probability distribution $p\left(x\right)$ and $p\left(y\right)$, and is defined as follows:

(11) $$MI(X,Y)=-\int_{x}\int_{y}f_{x,y}(x,y)\log\frac{f_{x,y}(x,y)}{f_{x}(x)f_{y}(y)}dxdy$$

When variables *X* and *Y* are completely independent or mutually independent, their mutual information is 0. The greater the mutual information between two variables, the stronger the correlation between them. In data processing, sensors with large mutual information with the target variable should be selected to improve the prediction of the algorithm. At the same time, features with small mutual information should be eliminated to reduce data redundancy, which is the so-called feature selection based on maximum correlation and minimum redundancy.

In the application of the mutual information method, the probability density estimation function can be used to approximate the marginal probability distribution to simplify the above formula:

(12) $$MI(X,Y)=-\frac{1}{N}\sum_{i=1}^{N}\log\frac{{\hat{f}}_{x,y}\left(x_{i},y_{i}\right)}{{\hat{f}}_{x}\left(x_{i}\right){\hat{f}}_{y}\left(y_{i}\right)}$$

where ${\hat{f}}_{x}\left(x_{i}\right)$ and ${\hat{f}}_{y}\left(y_{i}\right)$ are the joint probability distributions of X and Y, respectively, ${\hat{f}}_{x,y}\left(x_{i},y_{i}\right)$ is the joint probability density estimation function, and N is the number of data samples.

### 2.5. Operating Condition Identification Based on T-SNE

The idea of T-SNE is to convert the Euclidean distance between data points into conditional probability, and then use the conditional probability to measure the similarity between data points. The greater the similarity, the closer the distance between data points. The advantage of measuring the distance between samples in the form of probability is that in the process of mapping from high-dimensional space to low-dimensional space, although the distance between sample points may vary due to spatial differences, it can still maintain the relative distance between sample points, so that the originally distant sample points still maintain a relatively far distance. Specifically, for the sample point $x_{i}$, in the original high-dimensional space, its similarity to other sample points such as $x_{j}$ can be expressed by the conditional probabilities $p_{j/i}$ and $p_{i/j}$ of the Gaussian distribution as:

(13) $$p_{j/i}=\frac{\exp\left(-{\left\|x_{i}-x_{j}\right\|}^{2}/2\sigma_{i}^{2}\right)}{\sum_{k\neq i}\exp\left(-{\left\|x_{i}-x_{k}\right\|}^{2}/2\sigma_{i}^{2}\right)}$$

(14) $$p_{i/j}=\frac{\exp\left(-{\left\|x_{j}-x_{i}\right\|}^{2}/2\sigma_{j}^{2}\right)}{\sum_{k\neq j}\exp\left(-{\left\|x_{j}-x_{k}\right\|}^{2}/2\sigma_{j}^{2}\right)}$$

where *k* is the number of nearest neighbors of each point, $\sigma_{i}$ and $\sigma_{j}$ are the Gaussian variances of $x_{i}$ and $x_{j}$, respectively. Then, the symmetric joint probability distribution of data points in the original space is:

(15) $$p_{ij}=\frac{\left(p_{j/i}+p_{i/j}\right)}{2n}$$

where *N* is the size of the data. The variance σ in Formulas (13) and (14) is calculated by the binary search of perplexity $Perp\left(P_{i}\right)$, which is a manually defined parameter. Its function is to measure the number of points around each point in the effective domain that can form a smooth epidemic, and is defined as follows:

(16) $$Perp\left(P_{i}\right)=2^{H\left(P_{i}\right)}$$

where $H\left(P_{i}\right)$ is the Shannon entropy of $P_{i}$ and is defined as follows:

(17) $$H\left(P_{i}\right)=-\sum_{j}P_{j/i}\log_{2}P_{j/i}$$

In the low-dimensional space after dimensionality reduction, the mapping points of sample points $x_{i}$ and $x_{j}$ are $t_{i}$ and $t_{j}$, respectively. Assuming that the symmetric joint probability distribution between the two is represented by $q_{ij}$, it can be expressed as:

(18) $$q_{ij}=\frac{{\left(1+{\left\|t_{i}-t_{j}\right\|}^{2}\right)}^{-1}}{\sum_{k\neq l}{\left(1+{\left\|t_{k}-t_{l}\right\|}^{2}\right)}^{-1}}$$

where *k* and *l* respectively represent any two different points in a low dimensional space, defined by a t-distribution with a degree freedom of 1, which makes the points within the same cluster aggregate more tightly and the points in different clusters become more distant.

### 2.6. Reconstruction Error as Health Indicator

**Figure 4:**
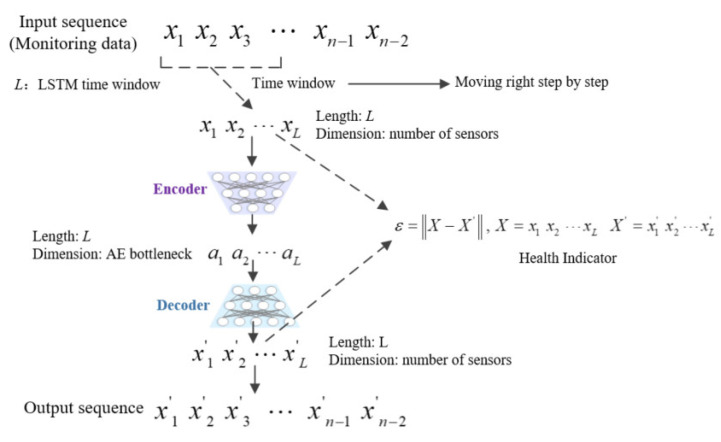
Workflow of HI construction based on LSTM-AE.

In the training process of LSTM-AE, we only use monitoring data of the initial state of the system, that is, the health status data of the system. Figure 4 shows the workflow of HI construction based on LSTM-AE, where *L* is the length of the LSTM time window, which is one of the hyperparameters of the model. For the monitoring data of the system, the time window with a length of *L* will gradually move to the right and intercept the time series with a length of *L* (the dimension of the time series depends on the number of sensors used). Then, the time series X is calculated by the decoder and encoder to obtain the reconstructed time series $X^{\prime }$.

As only normal data are used in the process of training the autoencoder, when the system state gradually deteriorates, the autoencoder model has poor reconstruction ability for the deviated monitoring data as it has not seen any data after state deviation. Therefore, the reconstruction error for the monitoring data of the system will gradually increase [39]. In the method proposed in this paper, the reconstruction error of system monitoring data using LSTM-AE will be used as the HI and is defined as follows:

(19) $$\varepsilon =\left\|X-X^{\prime }\right\|,\;X=x_{1}\;x_{2}\;\cdots x_{L}\;X^{\prime }=x_{1}^{\prime }\;x_{2}^{\prime }\cdots x_{L}^{\prime }$$

where *L* is the length of the LSTM time window, X is the input data, and $X^{\prime }$ is the data reconstructed by the autoencoder.

## 3. Experiments

To verify the effectiveness of the method proposed in this paper, the LSTM-AE-based HI construction method is validated using the C-MAPSS aircraft engine performance degradation simulation dataset published by NASA. The methods of sensor selection based on MI and T-SNE-based operating condition identification are applied. The effectiveness of the method is compared and analyzed with algorithms such as PCA and Vanilla-AE. All simulation experiments were carried out in a PyCharm 2021 (Community Edition) environment on a PC with an AMD Ryzen 7 3700X 8-core CPU and 16 GB memory.

### 3.1. C-MAPPS Datasets

**Figure 5:**
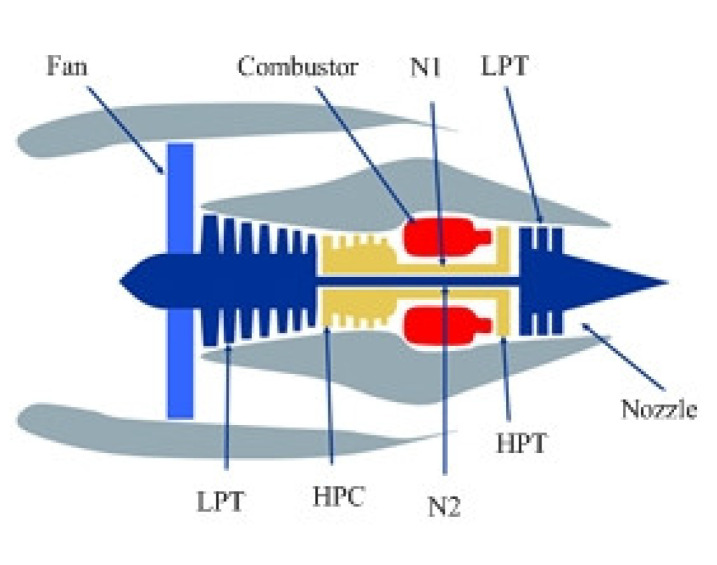
Structure diagram of a commercial modular aero-propulsion system.

As shown in Figure 5, a structure diagram of the aero-engine based on C-MAPSS contains several components such as a fan, combustion chamber, high/low pressure booster, turbine, and nozzle [40].

The C-MAPSS dataset consists of four sub-datasets (FD001 to FD004), each corresponding to different working conditions and fault modes. As shown in Table 1, FD001 and FD003 contain one operating condition, while FD002 and FD004 both contain six operating conditions. FD001 and FD002 have one fault mode, while FD003 and FD004 both have two fault modes. The number of engines in the datasets of FD001 to FD004 is 100, 260, 100, and 249, respectively. In these datasets, a total of 21 different sensors are used to measure the working condition, including physical information collected from different locations during the operating cycle of the aircraft engine (such as temperature, pressure, and speed).

**Figure 6:**
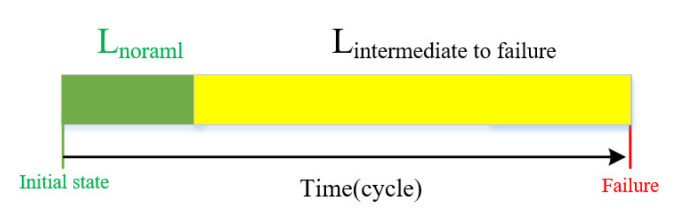
Data partition in the full lifecycle of engine.

During data processing, as shown in Figure 6, the data taken from the first 50 cycles of each engine were regarded as normal data, and only the normal data of each engine were used in the training of an autoencoder.

### 3.2. Data Preprocessing

Due to the different measurement intervals of different sensors, the data need to be normalized to make all sensor data ranges consistent, so as to achieve a better prediction effect. We adopt Z-score standardization to normalize the input data:

(20) $$X^{\prime }=\frac{X-\bar{X}}{\sigma }$$

where X is the time series data, $\bar{X}$ and σ are the average and variance of X, respectively, and $X^{\prime }$ is the normalized monitoring data.

**Figure 7:**
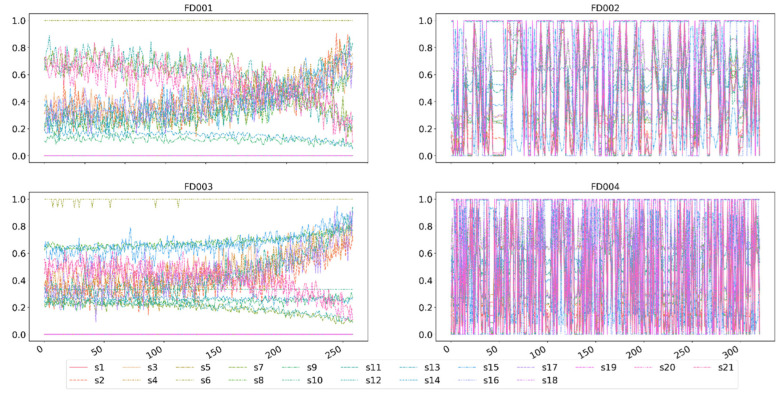
Normalized C-MAPSS sensor monitoring data from FD001 to FD004.

Figure 7 shows the normalized change trend of the original data of FD001, FD002, FD003, and FD004 in the C-MAPSS dataset. From Figure 4, it can be seen that the sensor data of FD001 and FD003 show a clear upward or downward trend, while FD002 and FD004 do not show see a clear pattern. The reason for this problem is that, as mentioned earlier, there are six different operating conditions in the FD002 and FD004 datasets. During the entire life cycle of the engine, the engine operates alternately between different operating conditions. For the situations of FD002 and FD004, the T-SNE method proposed in Section 2.5 should be used to prepare for the identification and division of such alternating operating conditions.

### 3.3. Sensor Selection

For the feature selection problem of time series based on mutual information, one of the difficulties is to find appropriate target variables [41]. For monitoring data, in most cases, the monotonicity and predictability of sensors cannot be guaranteed, nor can they fully reflect the health and degradation of the system. Therefore, it is not possible to randomly select a sensor signal as the target variable. To address this issue, we propose a time series target variable construction method based on PCA. Firstly, the PCA method is applied to reduce the dimensionality of multidimensional time series variables, and then the obtained principal components are smoothed. After that, the smoothed principal component is taken as the target variable of mutual information. In this way, the obtained target variable can represent the overall performance change of the system.

The mutual information correlation between different sensors and target variables is analyzed by taking engine 1 in the FD001 dataset as an example. It should be noted that some sensor signals in the monitoring data are constant values, which do not change with engine operation, such as s1, s5, s6, s10, s16, s18, and s19. After removing these data, a total of 14 sensor signals are obtained in the C-MAPSS dataset. Further, in order to reduce the redundancy between data, based on the feature selection idea of maximum correlation and minimum redundancy [42], before feature selection, some variables with high redundancy among themselves are removed according to the value of mutual information between variables. After the above selection, Table 2 shows the mutual information values of nine different sensors with the target variable.

It can be seen from Table 3 that the five sensors with high mutual information with the target variable are s7, s9, s11, s12, and s13, corresponding to total pressure at the high-pressure compressor outlet, physical core speed, static pressure at the high-pressure compressor outlet, ratio of fuel flow to Ps30, and corrected fan speed, respectively, as shown in Table 3.

### 3.4. Operating Condition Identification

The C-MAPSS datasets FD002 and FD004 contain six different operating conditions, which continuously alternate between each operating condition during the working process. In order to build a system HI, real-time and accurate identification of system operating conditions is necessary. We propose and apply the T-SNE method for real-time operating condition recognition of the monitoring data for datasets FD002 and FD004.

**Figure 8:**
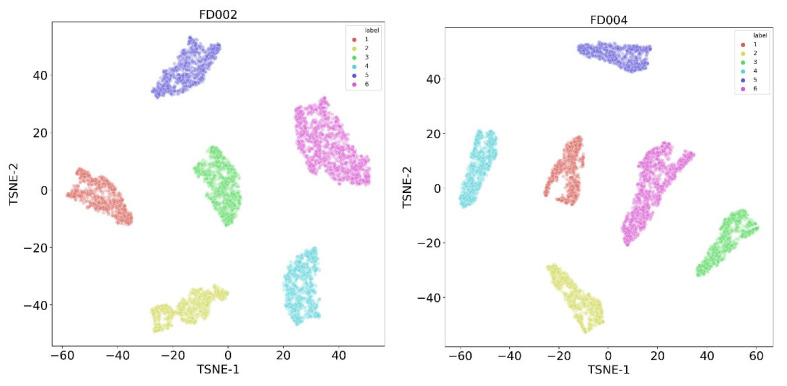
Clustering results of six operating conditions, with a sample size of 5000 cycles. Left-hand plot is FD002, and the right-hand plot is FD004.

We conduct operating condition identification on the FD002 and FD004 datasets based on the five sensor signals selected in Section 3.3. Figure 8 shows the effects of FD002 and FD004 using the T-SNE method for condition identification. From the graph, it can be seen that the data points of different operating conditions are effectively separated into different regions. The T-SNE method can accurately identify the engine operating conditions and preserve the general structure of the monitoring data. Thus, we can divide and normalize the data of FD002 and FD004 in Section 3.2, and eliminate the impact of variable operating conditions.

### 3.5. Performance Metrics

For HI construction, a set of metrics that can describe the quality of HI is needed, including monotonicity, trendability, and prognosability [43,44]. Among these, monotonicity represents the potential positive or negative trend of features. Assuming that the system does not undergo maintenance, it should be monotonic on the timeline for degraded features. The definition of monotonicity is as follows:

(21) $$monotonicity=\frac{1}{M}\sum_{j=1}^{M}\left|\sum_{k=1}^{N_{j}-1}\frac{\mathrm{sgn}\left(x_{j}(k+1)-x_{j}(k)\right)}{N_{j}-1}\right|$$

where M is the number of HIs, $N_{j}$ is the length of the *j*_th_ HI, $x_{j}(k+1)$ and $x_{j}(k)$ are the values of the *j*_th_ HI at time of k+1 and k, respectively. In PHM, the value of monotonicity is between 0 and 1, and the closer it is to 1, the more monotonic the predictor, which is more beneficial for prediction.

Trendability measures the similarity among the trajectories of the HI in multiple run-to-failure experiments. Generally, Pearson correlation is used to describe whether a set of HI has the same basic trend, and its calculation methods are as follows:

(22) $$trendability=\min_{j,k}\left|\mathrm{corr}\left(x_{j},x_{k}\right)\right|,\;j,k=1,\ldots ,M$$

where M is the number of HIs, $x_{j}$ and $x_{k}$ are the values of the *j*_th_ and *k*_th_ HI respectively, $\mathrm{corr}\left(\right)$ is the Pearson correlation function. When the lengths of $x_{j}$ and $x_{k}$ are different, interpolation processing is required. The value of trendability is between 0 and 1. The closer it approaches the value of 1, the better its predictability.

Prognosability reflects the dispersion of HI between faults and normal states, specifically:

(23) $$prognosability=\exp\left(-\frac{{\mathrm{std}}_{j}\left(x_{j}\left(N_{j}\right)\right)}{{\mathrm{mean}}_{j}\left|x_{j}(1)-x_{j}\left(N_{j}\right)\right|}\right),\;j=1,2,\cdots M$$

where M is the number of HIs, $N_{j}$ is the length of the *j*_th_ HI, $x_{j}$ is the value of the *j*_th_ HI, ${\mathrm{std}}_{j}$ is the standard deviation function, ${\mathrm{mean}}_{j}$ is the average function. The value of prognosability is between 0 and 1. The closer it is to 1, the better its predictability.

The fitness is the weighted sum of the three predictive indicators mentioned above:

(24) $$fitness=w_{m}\times Monotonicity+w_{t}\times Trendability+w_{p}\times Prognosability$$

where $w_{m}$, $w_{t}$, $w_{p}$ are the weight values, which are all equal to 1 in this paper.

### 3.6. Experimental Setting

To test the performance of the proposed model, in the CMAPSS dataset, normal operating data from FD001, FD002, FD003, and FD004 were selected, with 80% samples from each dataset being used for training, and the remaining 20% being used as testing data. Firstly, the number of neurons in the input layer of the model is determined by the input data. Due to the selection of five sensor signals in Section 3.3, the number of neurons in the input and output layers is five. The LSTM-AE model and Vanilla-AE neural network structure ultimately adopted in this article include an input layer, three encoding layers, an intermediate layer, three decoding layers, and an output layer. It should also be noted that LSTM-AE has an additional preprocessing step compared to Vanilla-AE. The purpose of this preprocessing is to reshape the input data into (samples, time steps, features). The network parameter settings are determined by the grid search method, where the shared bottleneck layer is a LSTM layer with eight nodes. A dropout layer with a dropout rate of 0.5 is employed after the shared bottom layer to prevent the model overfitting. The weights in the network are initialized randomly, and the model is trained using an Adam optimizer with a learning rate of 0.001. The final network structure, input size, and output size of each layer of LSTM-AE and Vanilla-AE, as well as the hyperparameters, are tabulated in Table 4, Table 5 and Table 6.

### 3.7. Health Indicator Evaluation

To verify the effectiveness of the proposed method, experiments were conducted on the four sets of data in C-MAPSS, FD001, FD002, FD003, and FD004. Among these, FD002 and FD004 contain six different operating conditions. Comparative analysis is conducted with PCA and Vanilla-AE, two widely used HI construction methods. Table 4 shows the performance of the three methods on the four datasets.

Table 7 shows the performance of the three methods on four datasets. We post the best results for each metric and dataset in bold font in Table 7. Firstly, for the two datasets FD002 and FD004, LSTM-AE outperforms PCA and Vanilla-AE in terms of monotonicity, trendability, prognosability, and fitness. For the FD001 and FD003 datasets, LSTM-AE outperforms PCA and Vanilla-AE in terms of monotonicity, but its trendability and prognosability are similar to, but slightly lower than, those of the PCA method, and both are higher than those of the Vanilla-AE method.

From Table 7, it can be seen that the LSTM-AE method has achieved better results than PCA and Vanilla-AE in terms of monotonicity, trendability, prognosability, and fitness. The exceptions are the two metrics of trendability and prognosability, which are slightly lower than those of the PCA method on the FD001 and FD003 datasets. In addition, we also notice that the PCA method performed well on the FD001 and FD003 datasets, but on the FD002 and FD004 datasets, the prognosability metric is almost equal to 0, which indicates that the PCA method has weak data processing ability for variable operating conditions.

**Figure 9:**
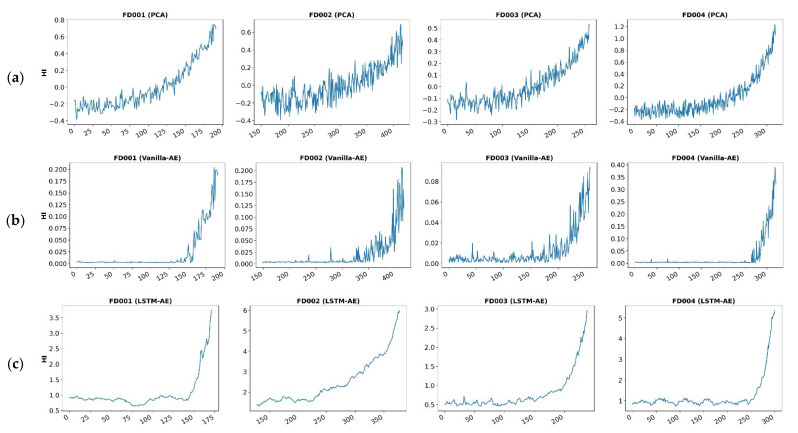
HIs built on PCA, Vanilla-AE, and LSTM-AE methods. (**a**) The first row is the PCA method applied to FD001, FD002, FD003, and FD004 respectively. (**b**) The second row is the Vanilla-AE method applied to FD001, FD002, FD003, and FD004, respectively. (**c**) The third row is the LSTM -AE method applied to FD001, FD002, FD003, and FD004, respectively.

In order to more intuitively demonstrate the predictive effects of the three methods, we visualize the HI curves of the engines obtained from the three methods in four datasets, as shown in Figure 9. From Figure 9, we can see that the PCA method has a certain upward trend in HI during the initial operation phase of the system during the long-life cycle, while Vanilla-AE and LSTM-AE are relatively stable in the initial stage. From the perspective of system degradation, the system has a good state in the initial stage, and HI should theoretically remain relatively stable at this time. Therefore, we believe that compared to PCA methods, AE-based methods can better reflect the degradation status of the system. On the other hand, comparing the HI curves of Vanilla-AE and LSTM-AE, it can be seen that due to the time series characteristics of monitoring data learned by LSTM neural networks, their HI curves are more stable and smoother compared to those of Vanilla-AE, thus having better predictive performance.

### 3.8. The Effect of LSTM Time Step on Predictive Performance

For the LSTM-AE model, time step is one of the most important hyperparameters and directly affects the prediction performance of HI construction. To analyze the impact of different time steps on the HI performance of the system, we compare the performance of the LSTM-AE model under different time steps. Table 8 shows the comparison of time steps of 5, 10, 15, 20, 25, and 30 cycles.

From Table 8, we can see that increasing the time step size can obviously improve the predictive performance of HI. When the time step size is 20 cycles, the proposed method achieves the highest number of optimal indicators on the four datasets FD001, FD002, FD003, and FD004, with item counts of 3, 4, 2, and 1, respectively. However, it should be noted that when using the proposed method for HI construction, the effect may deteriorate if the time step is too long. For example, when the step reaches 25 cycles, the predictive performance of various indicators decreases. The reason for this problem is that when the time step size is too large, the time series information of system degradation contained within a single step range is too much, which means the degradation information learned by the model becomes too vague. This is because a single time step may contain information such as the system operating normally, in addition to degradation or failure. Therefore, special attention should be paid to the hyperparameter of time step.

**Figure 10:**
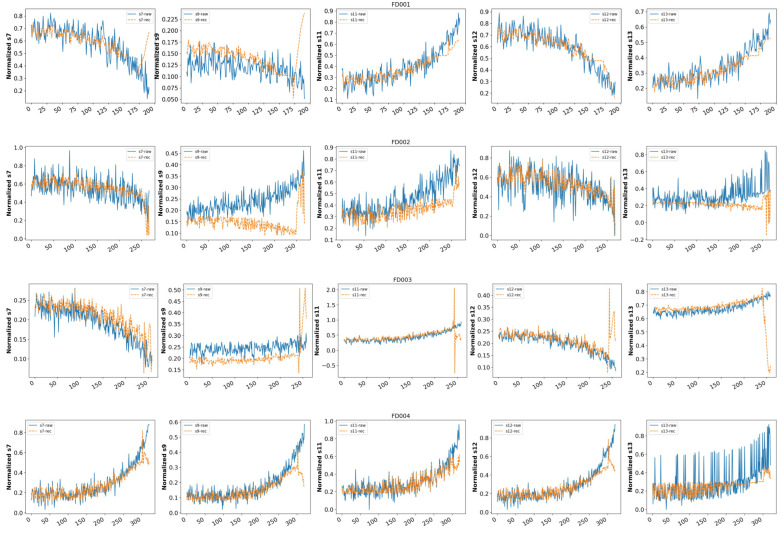
Raw and reconstructed sensor signals with LSTM-AE—s7, s9, s11, s12, and s13 of the CMAPSS dataset. The first row is the LSTM-AE method applied to FD001. The second row is the LSTM-AE method applied to FD002. The third row is the LSTM-AE method applied to FD003. The fourth row is the LSTM-AE method applied to FD004.

Figure 10 shows the sensor signals of the LSTM-AE model before and after AE reconstruction at a time step of 20 cycles. The reconstructed data are the s7, s9, s11, s12, and s13 sensor signals of the first engine in the FD001, FD002, FD003, and FD004 datasets. In the figure, the blue curve represents the original signal of the sensor, while the yellow curve represents the reconstructed signal. It can be seen from the figure that the amplitude of the reconstructed signal is basically smaller than that of the original signal.

In an ideal situation, the sensor signal reconstructed by the LSTM-AE model will basically overlap with the original signal in the initial stage. As the number of engine cycles increases, the engine will gradually deviate from its normal operating state, and the reconstructed signal will gradually deviate from the original signal. At this point, the reconstruction error, i.e., HI, will also gradually increase. Next, we analyze the specific situation of data reconstruction. Firstly, taking FD001 as an example, it can be seen from the signal fitting that when the engine is running at around 150 cycles, the reconstructed signal begins to generate a certain deviation, indicating that the engine begins to exhibit abnormalities at around 150 cycles and the engine’s performance begins to deteriorate. In addition, during the operation of the engine, due to different types of fault inputs in the simulation dataset, the system performance may deteriorate, causing some sensors’ signals to still be in normal working conditions, while others may experience significant deviations. For example, the s12 signal of FD002 has always had a good fitting effect during the process. We also notice that there is a certain deviation between the reconstructed signal of AE and the original signal for sensor signal s9. The reason for this phenomenon may be that S9 represents the physical core speed of the system, which fluctuates greatly and frequently during the engine operating cycle, and, at this time, the model’s reconstruction ability is limited.

## 4. Conclusions

In this paper, we propose an HI construction method based on an autoencoder and a long short-term memory neural network. The proposed method, LSTM-AE, is an unsupervised method as the model is fully trained based on the system’s normal operating monitoring data, and the abnormal data are not needed. The construction error of the AE model is adopted as the system HI. We built the overall framework for the monitoring data fusion and HI construction based on LSTM-AE and the data processing workflow, including techniques such as mutual information for sensor selection and t-distributed stochastic neighbor embedding for operating condition identification. Experiments were conducted based on the CMAPSS dataset to test the utility of the LSTM-AE on the construction of system HI. The LSTM-AE method showed better algorithm performance compared to PCA and Vanilla-AE models in the metrics of monotonicity, trendability, prognosability, and fitness. LSTM-AE proved capable of constructing an effective HI that can characterize the health state of the system. This ensures that the constructed HI is conducive to future analysis of failure prognostics, as well as RUL prediction, etc.

Regarding future work, we plan to apply our proposal in hybrid autoencoder architectures, such as deep adversarial autoencoders (AAEs), as AAEs can learn better representations and extract more robust features. Another direction of future work is to develop new indicators for prognostics; for example, instead of directly constructing an HI for prognostics, exploring indicators of feature importance that are interrelated for prognostics features might be promising.

## Figures and Tables

**Table 1:** Basic information of C-MAPSS dataset.

Datasets	FD001	FD002	FD003	FD004
Operating condition	1	6	1	6
Fault mode	1	1	2	2
Number of engines	100	260	100	249

**Table 2:** Mutual information between sensor signal and target variable.

Sensor Number	Value of MI	Units
s2	0.570	bit
s7	0.697	bit
s8	0.597	bit
s9	1.000	bit
s11	0.951	bit
s12	0.705	bit
s13	0.682	bit
s17	0.485	bit
s20	0.506	bit

**Table 3:** Selected engine sensors for HI construction.

Sensor Number	Description	Units
s7	Total pressure at High-Pressure Compressor outlet	kpa
s9	Physical core speed	rpm
s11	Static pressure at High-Pressure Compressor outlet	kpa
s12	Ratio of fuel flow to Ps30	—
s13	Corrected fan speed	rpm

**Table 4:** Neural network structure of LSTM-AE.

Model	Neural Network	Neuron Number
Input layer	LSTM	5
Encoder	LSTM	128
Encoder	LSTM	64
Encoder	LSTM	32
Middle layer	LSTM	8
Decoder	LSTM	32
Decoder	LSTM	64
Decoder	LSTM	128
Output layer	Dense	5

**Table 5:** Neural network structure of Vanilla-AE.

Model	Neural Network	Neuron Number
Input layer	Dense	5
Encoder	Dense	128
Encoder	Dense	64
Encoder	Dense	32
Middle layer	Dense	8
Decoder	Dense	32
Decoder	Dense	64
Decoder	Dense	128
Output layer	Dense	5

**Table 6:** Hyperparameters of neural networks.

Hyperparameters	Value
Batch size	128
Learning rate	0.001
Optimizer	Adam
Loss	Mae
Epochs	200
Dropout rate	0.5

**Table 7:** Performance results of PCA, Vanilla-AE, and LSTM-AE for FD001, FD002, FD003, and FD004 (best results for each metric and dataset are presented in bold font).

Predictor	FD001				FD002			
	Mono	Tren	Prog	Fitness	Mono	Tren	Prog	Fitness
PCA	0.335	**0.892**	**0.855**	2.082	0.194	0.0004	0.751	0.9454
Vanilla-AE	0.246	0.796	0.818	1.860	0.142	0.419	0.617	1.178
LSTM-AE	**0.449**	0.890	0.841	**2.180**	**0.446**	**0.861**	**0.822**	**2.129**
Predictor	FD003				FD004			
	Mono	Tren	Prog	Fitness	Mono	Tren	Prog	Fitness
PCA	0.330	**0.805**	**0.701**	**1.836**	0.169	0.0002	0.581	0.7502
Vanilla-AE	0.215	0.640	0.496	1.351	0.15	0.383	0.529	1.062
LSTM-AE	**0.419**	0.718	0.605	1.742	**0.272**	**0.387**	**0.641**	**1.300**

monotonicity—mono, trendability—tren–, prognosability—prog, and fitness.

**Table 8:** Results of the performance of Vanilla-AE for time step of 5, 10, 15, 20, and 25 cycles (best results for each metric and dataset are presented in bold font).

Time step (cycle)	FD001				FD002			
	Mono	Tren	Prog	Fitness	Mono	Tren	Prog	Fitness
5	0.298	0.880	**0.852**	2.030	0.184	0.356	0.537	1.077
10	0.313	0.801	0.739	1.853	0.296	0.776	0.657	1.729
15	0.406	0.709	0.795	1.910	0.358	0.791	0.648	1.797
20	**0.449**	**0.890**	0.841	**2.180**	**0.446**	**0.861**	**0.822**	**2.129**
25	0.448	0.886	0.817	2.151	0.358	0.772	0.729	1.859
Time step (cycle)	FD003				FD004			
	Mono	Tren	Prog	Fitness	Mono	Tren	Prog	Fitness
5	0.329	0.845	0.561	1.735	0.216	0.371	0.549	1.136
10	0.383	0.860	0.330	1.573	0.284	0.454	0.610	**1.348**
15	0.418	0.707	0.545	1.670	0.316	**0.406**	0.614	1.336
20	0.419	0.718	**0.605**	**1.742**	0.272	0.387	**0.641**	1.300
25	**0.448**	**0.820**	0.441	1.709	**0.323**	0.188	0.453	0.964

monotonicity—mono–, trendability—tren–, prognosability—prog, and fitness.

## Data Availability

Data sharing is not applicable to this article as no new data were created or analyzed in this study.
